# Critical Role of IRF-5 in the Development of T helper 1 responses to *Leishmania donovani* infection

**DOI:** 10.1371/journal.ppat.1001246

**Published:** 2011-01-06

**Authors:** Andrea Paun, Rashmi Bankoti, Trupti Joshi, Paula M. Pitha, Simona Stäger

**Affiliations:** 1 Sidney Kimmel Comprehensive Center, The Johns Hopkins University, Baltimore, Maryland, United States of America; 2 Department of Molecular and Comparative Pathobiology, The Johns Hopkins School of Medicine, Baltimore, Maryland, United States of America; 3 Biology Department, The Johns Hopkins University, Baltimore, Maryland, United States of America; Imperial College London, United Kingdom

## Abstract

The transcription factor Interferon Regulatory Factor 5 (IRF-5) has been shown to be involved in the induction of proinflammatory cytokines in response to viral infections and TLR activation and to play an essential role in the innate inflammatory response. In this study, we used the experimental model of visceral leishmaniasis to investigate the role of IRF-5 in the generation of Th1 responses and in the formation of Th1-type liver granulomas in *Leishmania donovani* infected mice. We show that TLR7-mediated activation of IRF-5 is essential for the development of Th1 responses to *L. donovani* in the spleen during chronic infection. We also demonstrate that IRF-5 deficiency leads to the incapacity to control *L. donovani* infection in the liver and to the formation of smaller granulomas. Granulomas in *Irf5^-/-^* mice are characterized by an increased IL-4 and IL-10 response and concomitant low iNOS expression. Collectively, these results identify IRF-5 as a critical molecular switch for the development of Th1 immune responses following *L. donovani* infections and reveal an indirect role of IRF-5 in the regulation of iNOS expression.

## Author Summary

*Leishmania donovani* is a parasite that currently infects 12 million people around the world. In order to better understand why this parasite causes incurable disease we chose to investigate how the immune system sees *L. donovani*. The immune system sees infecting organisms by the recognition of molecules that are specifically expressed by pathogens. This is done by a family of receptors expressed by cells called Toll Like Receptors (TLRs). When TLRs recognize a pathogen it leads to a molecular chain reaction within the cell resulting in the release of cytokines. Interferon Regulatory Factors (IRFs) are a very important part of this signaling chain. The protein we have studied, IRF-5, has been identified as having a key role in inducing pro-inflammatory cytokines following the recognition of viruses by TLRs. However, whether it plays a role in the immune response to parasitic disease has not yet been examined. In this study we infected mice deficient of IRF-5 with *L. donovani* and demonstrate for the first time that IRF-5 is essential to develop a protective response against this parasite. These results are important as they help us to understand the molecular mechanisms required for an immune response to fight *L. donovani*.

## Introduction

The protozoan parasite *Leishmania donovani* is the causative agent of visceral leishmaniasis (VL), a chronic life threatening disease if untreated. In the experimental model of VL, the two main target organs are the liver and the spleen [1]. While the spleen stays chronically infected, infection in the liver is self-resolving within 6-8 weeks due to the development of a Th1-dominated granulomatous response, which is characterized by high IFNγ production. This response is induced by IL-12 secreted by dendritic cells (DC) [2], [3], [4] and is crucial for parasite control and disease resolution in the liver, together with TNFα production and expression of inducible nitric oxide synthase (iNOS) by macrophages [1]. Studies using *Myd88^-/-^* mice have highlighted the importance of toll like receptors (TLRs) in the induction of IL-12 production by DC and the development of Th1 immune responses in *Leishmania* infection [5]. More recently, TLR9 has been shown to be required for IL-12 production by DC in a model of cutaneous leishmaniasis [6], [7] and also in *Trypanosoma cruzi* infected mice [8]. However, in contrast to *T. cruzi* infections, TLR9 deficiency in mice infected with *L. major* did not prevent the development of Th1 responses and only resulted in a transient disease exacerbation [6], [9]. As MyD88^-/-^ mice are highly susceptible to *Leishmania* infection [5], this suggests that in addition to TLR9, other TLRs as well as IL-1 and IL-18 may also be involved in the generation of Th1 responses and in the induction of host protective immunity. Since *Leishmania* parasites reside in the phagolysosomes of the host cells, other endosomally localized TLRs, such as TLR 7 and 8 could be involved in the recognition of this pathogen [10], [11].

Interferon Regulatory Factor 5 (IRF-5) has been shown to be involved in the transcriptional activation of both Type I IFN genes and genes encoding key proinflammatory cytokines such as IL-12, TNFα and IL-6 [12], [13], [14], [15]. This transcription factor can be activated by TLR7 and TLR9 via the MyD88 signaling pathway and/or directly by viral infections and Type I interferon [16]. In vivo, IRF-5 has been shown to play a role in the innate antiviral immune response. Indeed, lack of IRF-5 expression in genetically modified *Irf5^-/-^* mice resulted in attenuation of Type I IFN, TNFα and IL-6 production in response to viral infection [13], [17], [18]. However, the antiviral effect of IRF-5 deficiency appeared to be cell type specific and mainly affected DCs and plasmacytoid DCs (pDCs), rather than macrophages [16], [17]. More recently, IRF-5 was also shown to cooperate with, among others, NOD2 and TBK1 in triggering expression of Type I interferon in response to *Mycobacterium tuberculosis* [19].

The aim of this study was to examine whether IRF-5 also plays a role in the regulation of the immune response to parasitic infections. Here we demonstrate that IRF-5 deficiency results in severe impairment in the development of Th1 immune responses following *L. donovani* infection. Moreover, *Irf5^-/-^* mice failed to develop typical Th1-type granulomas and to control infection in the liver, demonstrating a vital role for IRF-5 in the induction of the anti-parasitic response.

## Results

### IRF-5 is required for disease control in the liver

**Figure 1:**
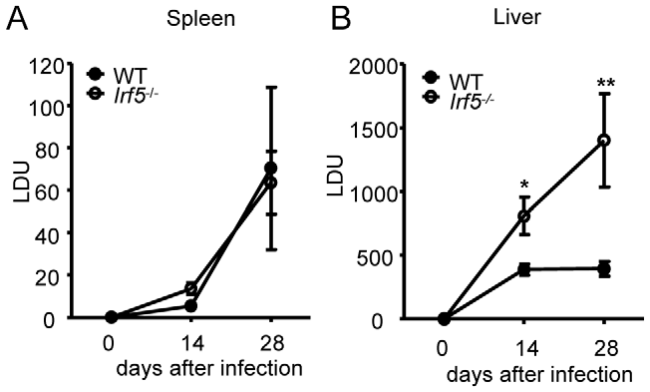
*Leishmania donovani* infection in *Irf5* ^-/-^ mice. Parasite burdens in the spleen (A) and liver (B) were determined for infected WT and *Irf5* ^-/-^ mice at d14 and d28 pi as described. Data shown is the mean ± SEM and is representative of two independent experiments. * denotes *p*<0.05 and ** denotes *p*<0.01.

The transcription factor IRF-5 is an important downstream regulator of the TLR/MyD88 signaling pathway and is involved in the induction of several key proinflammatory cytokines [13], [16], [17]. As TLRs have been implicated in the recognition of *Leishmania* parasites [5], [6], [7], [20], [21], [22], [23], we first wanted to assess whether IRF-5 was at all involved in the generation of protective immunity against *L. donovani*. Hence, we infected wild type (WT) and *Irf5^-/-^* mice and monitored the course of infection at several time points. We observed similar parasite burdens for WT and *Irf5^-/-^* mice in the spleen (Fig. 1A). In contrast, the disease was exacerbated in the livers of *Irf5^-/-^* mice, with a parasite burden of approximately two times higher at d14 pi and almost 4-fold higher than in the WT controls by d28 pi (Fig. 1B). These data suggest that the requirements for IRF-5 in the immune response to *L. donovani* are strictly organ specific, at least until day 28p.i..

### IRF-5 deficiency results in defective Th1 responses

**Figure 2:**
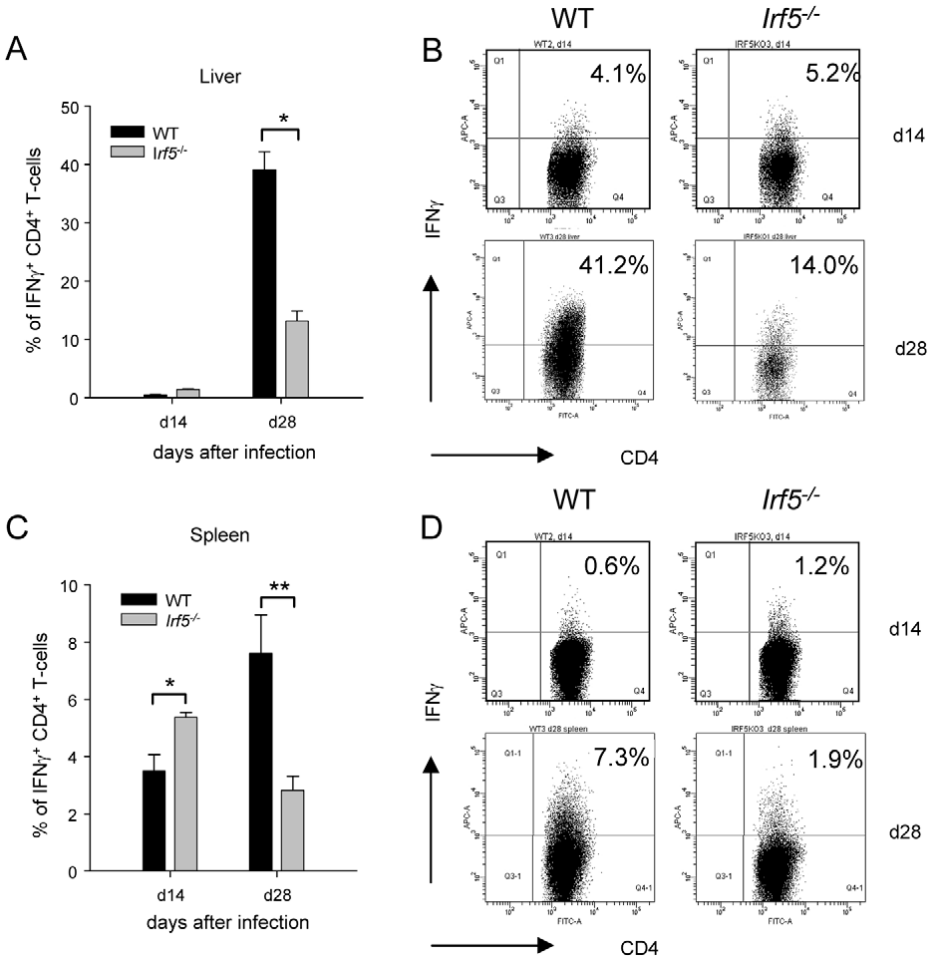
*Irf5* ^-/-^ mice have a defective Th1 response. The percentage of CD4^+^ T cells producing IFNγ as a response to *L. donovani* infection in the liver (A) and the spleen (C) of WT and *Irf5* ^-/-^ mice was determined by intracellular flow cytometry. Representative scatter plots from WT and *Irf5* ^-/-^ mice infected with *L. donovani* showing IFNγ production by CD4^+^ T cells in the liver (B) and spleen (D) at different times p.i.. Data is shown as the mean ± SEM. Flow cytometry data is representative of two independent experiments. * denotes *p*<0.05 and ** denotes *p*<0.01.

As IFNγ-producing CD4^+^ T helper cells are crucial for the control of *L. donovani* infection in the liver, we next investigated whether these responses were being generated in *Irf5^-/-^* mice. IFNγ–producing CD4^+^ T cells were already detected at d14 of infection in both the liver and spleen of WT mice, although still at low frequency. The frequency of IFNγ^+^ CD4^+^ T cells increased in the later stages of infection and by d28 pi, 39.1% of CD4^+^ T cells in the liver (Fig. 2A and B) and 7.6% in the spleen (Fig. 2C and D) of WT mice were producing IFNγ. In marked contrast, the generation of IFNγ-producing CD4^+^ T cells was significantly impaired in the livers of *Irf5^-/-^* mice (Fig. 2A), where only 13.2% of CD4^+^ T cells were IFNγ^+^ at d28 (a 60% reduction), and in the spleen (Fig. 2C), where only 2.8% of CD4^+^ T cells were found to be secreting IFNγ (a 60% reduction compared to WT controls). Interestingly, at day 14p.i., the frequency of IFNγ^+^ CD4^+^ T-cells in the liver and spleens of *Irf5^-/-^* mice was comparable, if not better than in WT mice (Fig. 2). This suggests that initially Th1 responses are being generated in the absence of IRF-5, however this transcription factor is essential for the maintenance and further expansion of Th1 responses.

### *Irf5^-/-^* mice fail to develop Th1-type granulomas

**Figure 3:**
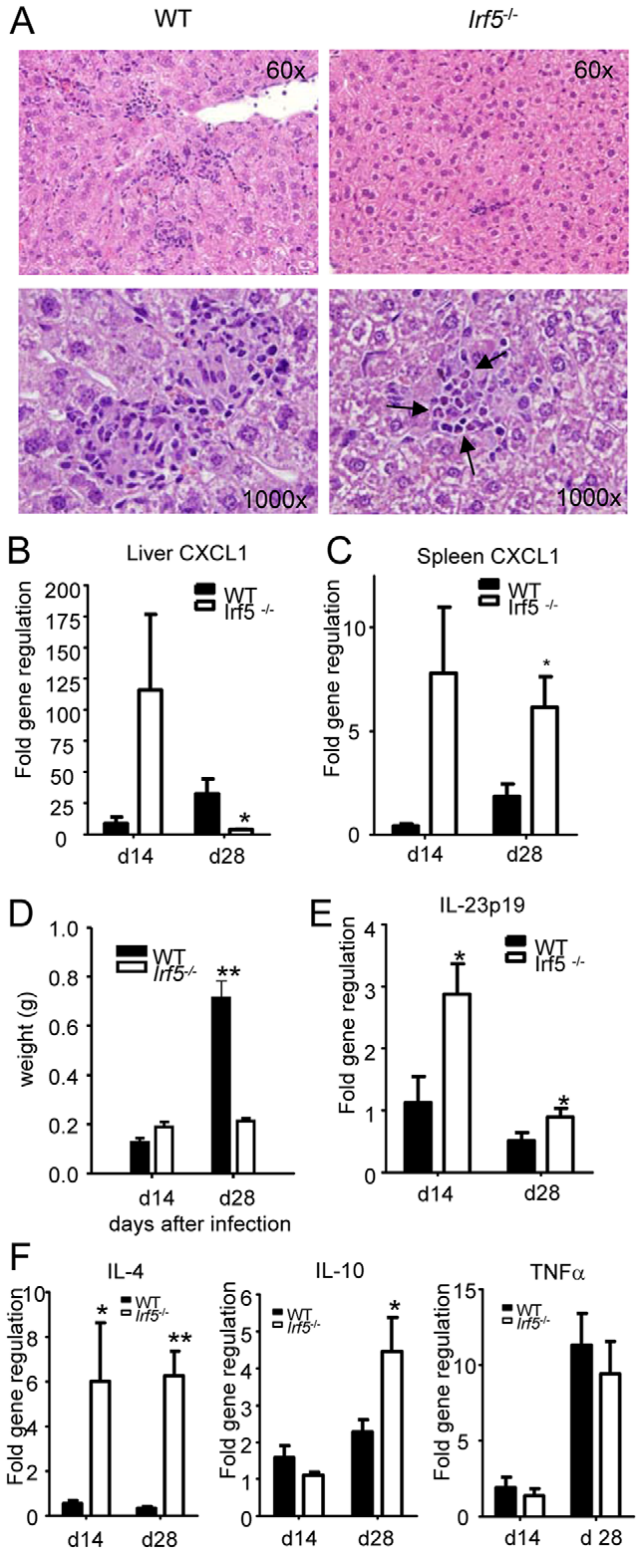
*Irf5* ^-/-^ mice do not develop Th1-type granulomas. (A) Representative granulomas from H&E stained liver sections at d28 pi in WT and *Irf5* ^-/-^ mice. Pictures were taken at the indicated magnifications and arrows indicate the presence of neutrophils in the granulomas. (B, C) Real time PCR analysis of the livers (B) and spleen (C) measuring mRNA for CXCL1. (D) Spleen weights at d14 and d28 pi. The body weight of C57BL/6 WT and *Irf5^-/-^* mice was comparable. (E) Real time PCR analysis of the livers from infected mice measuring mRNA for IL-23 p19. (F) Real time PCR measurement of cytokine mRNA in the livers of infected mice indicates a skewing toward a Th2 environment. All data is presented as the mean ± SEM combined from two independent experiments. * denotes *p*<0.05 and ** denotes *p*<0.01.

In order to determine whether the defective generation of Th1 responses detected in *Irf5^-/-^* mice had an impact on the Th1-type granuloma formation normally observed in the livers of *L. donovani* infected WT mice [1], we proceeded to analyze H&E stained sections of livers from WT and *Irf5^-/-^* mice. Despite an approximately 4-fold higher liver parasite burden in *Irf5^-/-^* mice (Fig. 1B), the number of granulomas/inflammatory foci observed in *Irf5^-/-^* mice at d28 p.i. was reduced approximately by 77% compared with WT mice (406.7±15.7 granuloma/100 microscopic fields in WT mice vs. 92±38 granuloma/100 microscopic fields in *Irf5^-/-^* mice). A closer histological analysis also revealed that the granulomas in *Irf5^-/-^* mice were smaller in size and had a different cellular composition, characterized by a marked infiltration of polymorphonucleated cells that were identified as neutrophils (Fig. 3A). Neutrophils were also observed at higher frequencies in the spleens of infected *Irf5^-/-^* mice compared to WT controls (Fig. S1 A and B). Increased neutrophil infiltration correlated with higher mRNA levels for CXCL1, a chemokine receptor known for its neutrophil chemoactractant activity, in the liver (day 14 p.i. only) and spleens of *Irf5^-/-^* compared to WT mice (Fig. 3 B and C). Of additional interest is the observation that the mononuclear cell infiltrates in the livers of *Irf5^-/-^* mice were significantly reduced at day 28 p.i. compared to WT mice (data not shown and Fig. 2B). Moreover, in *Irf5^-/-^* mice we could not observe any splenomegaly, which is a typical symptom of VL in WT mice (Fig. 3D). These results suggest that IRF-5 is an essential factor in the maintenance of the inflammatory responses generated during *L. donovani* infection. IL-23 has recently been shown to be involved in immune cell homing to infected target cells [24], hence we also assessed whether this cytokine was expressed at different levels in the livers of *Irf5^-/-^* mice. To our surprise, IL-23 p19 was slightly upregulated in *Irf5^-/-^* mice at day 14 p.i., but was induced at similar levels at later stages of infection in both WT and *Irf5^-/-^* mice (Fig. 3E).

Since *Irf5^-/-^* granulomas were not the typical Th1-type granulomas which develop in infected WT mice and, unlike WT mice, *Irf5^-/-^* mice failed to eliminate the parasites, we next examined the cytokine environment in the livers of both groups of mice. IRF-5 deficiency resulted not only in a very weak IFNγ responses (Fig. 2B), but also in a significantly higher expression of IL-4 and IL-10 mRNA compared to WT mice (Fig. 3 F). Interestingly, the differences in cytokine mRNA levels were observed only at d28 pi but not during the first 2 weeks of infection. Surprisingly, however, we did not observe any significant differences in the relative levels of TNFα (Fig. 3E), IL-13, or IL-5 (data not shown) between the two groups. Moreover, *Irf5^-/-^* and WT mice showed comparable frequencies of IL-10- and IL-17-producing CD4^+^ T cells at d28 pi (data not shown). Taken together, these data suggest that unlike in WT mice, *L. donovani* infection in *Irf5^-/-^* mice induces a very small inflammatory infiltration in the liver and results in the generation of an IL-4-dominated response, resulting in a failure to control parasite growth.

### Attenuation of IL-12p35 expression in *L. donovani* infected *Irf5^-/-^* mice

**Figure 4:**
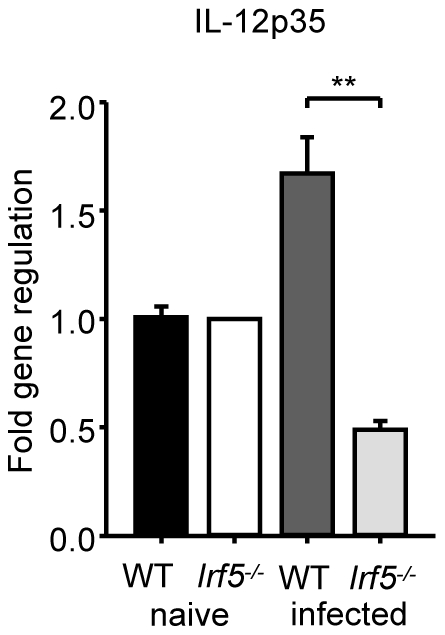
*L. donovani* fails to induce IL-12p35 expression in *Irf5^-/-^* mice. WT and *Irf5* ^-/-^ mice were infected with *L. donovani* amistigotes. At 5 h, splenic cDCs were purified. IL-12p35 mRNA was detected by real time PCR. Data is shown for n = 3 WT samples and n = 4 *Irf5* ^-/-^ samples.* denotes *p*<0.05 and ** denotes *p*<0.01.

It is well established that the production of IL-12 by DCs is crucial for the development of Th1 cells [25], although an IL-12-independent mechanism for the induction Th1 responses has also been described [26]. A study in *Myd88^-/-^* mice infected with *L. major* has highlighted the importance of TLRs, IL-1 and/or IL-18 in the induction of IL-12 and the generation of Th1 responses; MyD88 deficiency also resulted in complete abrogation of IFNγ production by CD4^+^ T cells and an inability to control infection [5]. More recently, TLR9 activation has been shown to play a crucial role in the induction of IL-12 secretion by DCs following *T. cruzi* and *Leishmania* infections [6], [7]. As the development of IFNγ-producing CD4^+^ T cell in *L. donovani* infected mice was shown to depend upon IL-12 production by conventional CD11c^hi^ DC (cDC), we examined whether IRF-5 deficient DC were able to produce IL-12 *in vivo* following *L. donovani* infection. In agreement with the literature [27], we detected a moderate increase in IL-12p35 mRNA levels in cDCs isolated from infected WT mice (Fig. 4). However, induction of IL-12p35 was not detected in IRF-5 deficient cDCs. In contrast, IL-12p40 mRNA expression was induced in cDCs of Irf5^-/-^ at similar levels as in cDC of WT mice (data not shown). Since the p40 component of IL-12 is shared with IL-23, the upregulation of p40 may be caused by an increased expression of IL-23 (s. Fig. 3E).

### IRF-5 indirectly regulates iNOS expression

**Figure 5:**
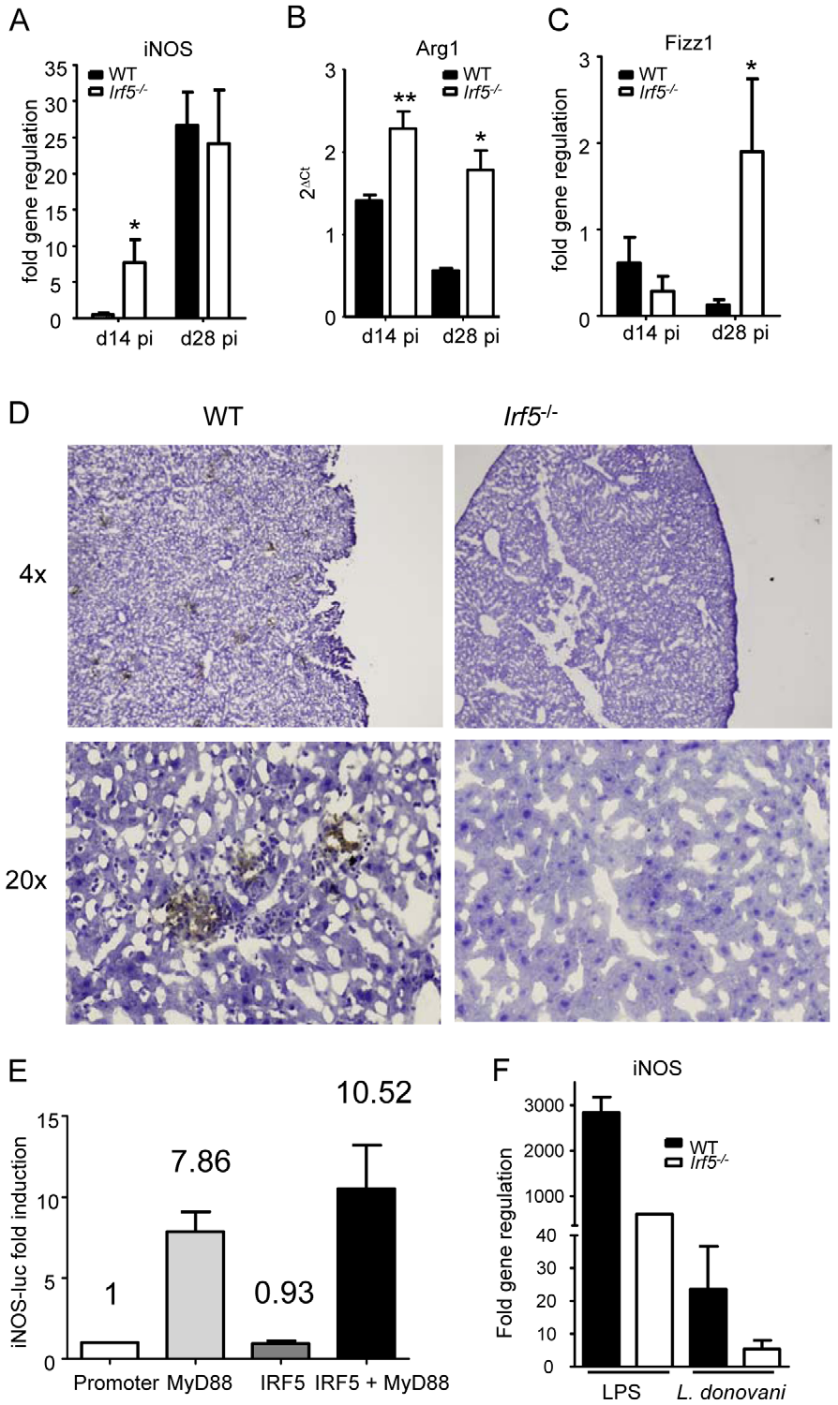
IRF-5 deficiency results in defective iNOS production. Real time PCR analysis of livers from infected mice measuring mRNA for iNOS (A), arginase1 (B) and Fizz1 (C). All data is presented as the mean ± SEM combined from two independent experiments, n = 6-9 mice per group. (D) iNOS protein in the liver was examined by immunohistochemistry. (E) Induction of the *NOS2* promoter by MyD88 and IRF-5 was measured by luciferase assay. Data shown is fold induction of the promoter compared to promoter only controls and represents the mean± SEM for triplicate samples from 2 independent experiments. * denotes *p*<0.05 and ** denotes *p*<0.01. (F) Real time PCR analysis of iNOS mRNA in WT and Irf5-/- macrophages stimulated with LPS and/or *L.donovani* amastigotes. Data is shown for n = 3.

Inducible nitric oxide synthase (iNOS) is a key enzyme involved in the production of nitric oxide (NO), which has direct microbial toxicity and is also involved in the regulation of cytokine gene expression and cytokine responsiveness. NO is typically produced by classically activated macrophages upon triggering of IFN and TLR pathways that enhance the expression of iNOS [28], [29], [30]. In the absence of a strong IFNγ response and the presence of an IL-4 dominated immune response in infected *Irf5^-/-^* mice, we were curious as to whether iNOS (*NOS2*) was still able to be induced at all in *Irf5^-/-^* mice. To our surprise, we found no difference in the induction of iNOS mRNA in the livers of *Irf5^-/-^* and WT mice (Fig. 5A). As the absence of IRF-5 appeared to be leading to a Th2-like state in the liver, we were also interested in determining whether markers for the alternative activation of macrophages were being induced in mice deficient in IRF-5. Using real time PCR we only found a slight increase in the induction of *Arg1* (Fig. 5B) and *Fizz1* (Fig. 5C) in the livers of infected *Irf5^-/-^* mice compared to WT mice.

Since iNOS mRNA levels were similar in *Irf5^-/-^* and WT mice, we next investigated whether this message was being translated into protein. iNOS protein was expressed in the livers of infected WT mice, mainly in granulomas (Fig. 5D). In contrast, it was not detected in infected *Irf5^-/-^* mice at any time point after infection (Fig. 5D).

To determine whether IRF-5 directly regulates transcription of *iNOS*, we used a luciferase reporter assay, where luciferase was under the control of the *NOS2* promoter cotransfected with MyD88 and/or IRF-5 expression plasmids. As shown in Figure 5E and in agreement with the literature [28], [30], MyD88 expression induced a 7.86 fold increase in luciferase activity. In contrast, IRF-5 failed to induce luciferase activity, suggesting that overexpression of inactivated IRF-5 alone does not stimulate the transcriptional activity of the *NOS2* promoter. Co- transfection with both MyD88 and IRF-5 expression plasmids resulted only in a slight increase in the transcriptional activity of the *NOS2* promoter. Taken together, these results indicate that IRF-5 does not stimulate transcription of iNOS but may play a role to indirectly regulate the iNOS response to *L. donovani* at the posttranscriptional level.

We next infected WT and *Irf5^-/-^* bone marrow-derived macrophages in vitro with *L. donovani* and monitored the iNOS mRNA expression by real time PCR. In agreement with the results shown in Fig. 5E, IRF-5 deficiency did not affect the iNOS mRNA level (Fig. 5F) or the NO production (Fig. S2 A) following *L. donovani* infection, suggesting that IRF-5 does not directly induce transcription of iNOS. However, when we stimulated WT and *Irf5^-/-^* macrophages with LPS, we could see a marked decrease in both iNOS mRNA transcipts (Fig. 5F) and NO production (Fig. S2 A). This was possibly caused by a defective pro-inflammatory response in *Irf5^-/-^* macrophages (Fig. S2 B). This defect was not observed in *Irf5^-/-^* macrophages infected with *L. donovani* (Fig. S2 B), suggesting that the induction of pro-inflammatory cytokines in macrophages infected in vitro with *L. donovani* is IRF-5 independent. This data implies that the inflammatory signals downstream of TLR triggering largely contribute to the induction of iNOS. Thus it is possible that the defective pro-inflammatory response observed in *L. donovani* infected *Irf5^-/-^* mice (Fig. 2 and 3) is mainly responsible for the reduction in iNOS expression in these mice.

### IRF-5 is upregulated in T-cells during *L. donovani* infection

**Figure 6:**
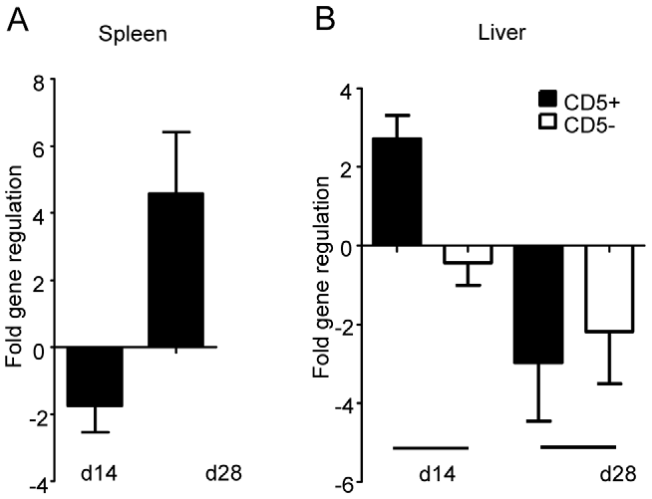
IRF-5 is upregulated in T-cells during *L. donovani* infection. Real time PCR analysis of spleens (A) and livers (B) from infected WT mice measuring mRNA for IRF-5. All data is presented as the mean ± SEM combined from two independent experiments, n = 6 mice per group.

Since IRF-5 seems to be involved in the maintenance of the inflammatory response to *L. donovani* and in the development of protective Th1-responses, we were interested in which cells IRF-5 expression is essential for displaying and/or inducing anti-leishmanial effector function. Thus, we analyzed the cell-specific expression pattern of IRF-5 mRNA in the liver and the spleen at different time points during infection (Fig. 6). Interestingly, in the spleen IRF-5 mRNA was only upregulated in the CD5^+^ population, corresponding to T-cells. The increase in IRF-5 mRNA levels was only detected at d28 p.i. (Fig. 6A). Splenic B-cells, macrophages, dendritic cells, and neutrophils did not show increased expression of IRF-5 mRNA at any time point analyzed (data not shown). In the liver, only the CD5 positive fraction (T-cells) had upregulated IRF-5 mRNA at d14 p.i. (Fig. 6B). However by d28 p.i., only few T-cells were present in the liver (Fig. 2 and 3) and at this time point T-cells did not express IRF-5 mRNA at levels greater than in naïve mice.

### TLR7 is essential for the development of host protective Th1 responses

We and others have previously shown that IRF-5 can be activated by TLR7 [16] and TLR9 [13] via the MyD88 signaling pathway. Indeed, it was recently shown that *Leishmania* infections in *Tlr9^-/-^* mice induced transiently deficient Th1 responses [7], [9], suggesting that the regulation of these responses in *Leishmania* infections might also be governed by other pathogen recognition pathways. Thus, we next investigated which TLR was involved in the activation of IRF-5 and consequently in the modulation of Th1 responses following *L. donovani* infection.

**Figure 7:**
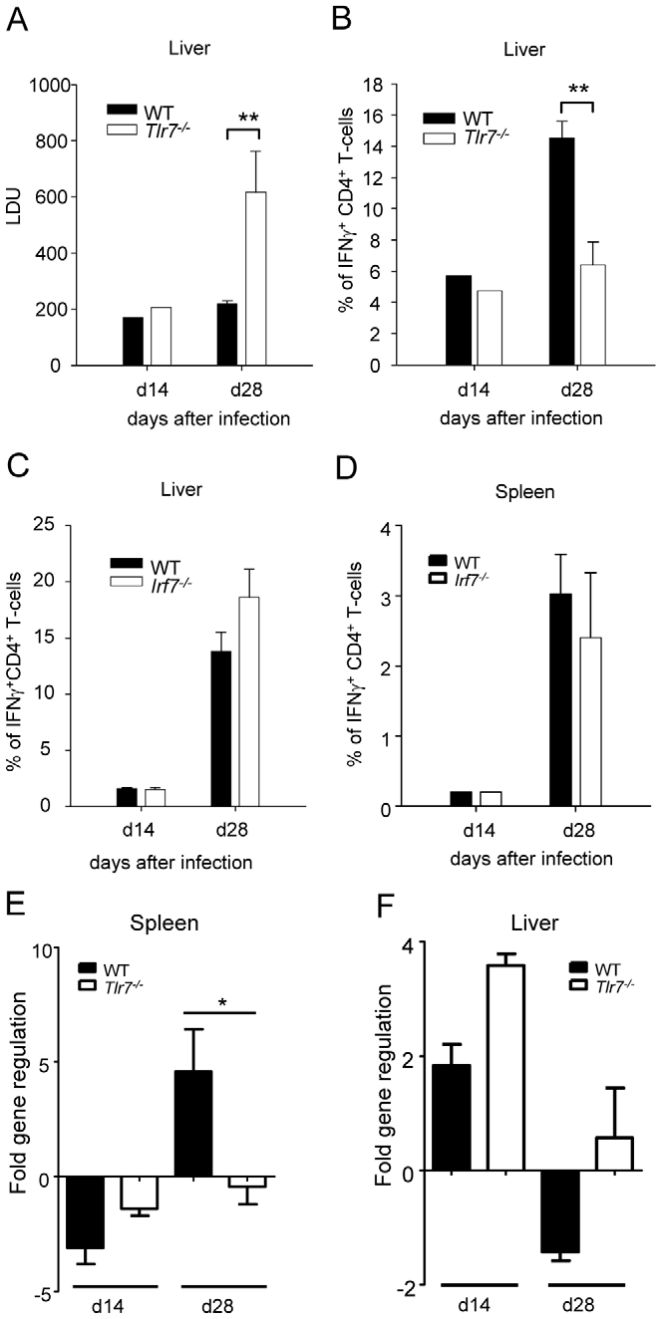
TLR7 activation is essential for the development of Th1 responses following *L. donovani* infection. (A) The hepatic parasite burden was determined for infected WT and *Tlr7* ^-/-^ mice at d14 and d28 pi as described. (B) The percentage of CD4^+^ T cells producing IFNγ as a response to *L. donovani* infection in the liver of WT and *Tlr7* ^-/-^ mice was determined by intracellular flow cytometry. (C) Percentage of IFNγ^+^ CD4^+^ T-cells in the liver and (D) the spleen of WT and *Irf7^-/-^* mice. (E,F) Real time PCR analysis of spleens (E) and livers (F) from infected WT and *Irf7^-/-^* mice measuring mRNA for IRF5. Data is shown as the mean ± SEM. Data is representative of two independent experiments.

Hence we proceeded to assess whether TLR7 was involved in the recognition of *L. donovani* and in the generation of parasite-specific Th1 responses. To our surprise, *L. donovani* infection in *Tlr7^-/-^* mice resulted in a approximately 3-fold higher hepatic parasite burden at day 28pi compared to WT mice (Fig. 7A); as seen in *Irf5^-/-^* mice, the splenic parasite burden in *Tlr7^-/-^* mice was similar to the WT control group (data not shown). These results suggest that TLR7 plays a role in the recognition of *L. donovani* parasites and in the generation of protective immune responses against *Leishmania*.

We then determined the frequency of IFNγ^+^ CD4^+^ T-cells in infected *Tlr7^-/-^* mice and WT controls. At day 14 p.i the frequency of IFNγ producing CD4^+^ T-cells was similar in the liver of *Tlr7^-/-^* mice compared to the WT control group. However, at day 28, only 6.4% of CD4^+^ T-cells in the liver of *Tlr7^-/-^* mice was producing IFNγ compared to 14.5% in WT mice (Fig. 7B and S3). A similar reduction in IFNγ production was observed in the spleen (data not shown). Interestingly, the extent of the inflammatory cell infiltration in the livers of *Tlr7^-/-^* mice at day 28 p.i. was comparable to WT mice (data not shown). Taken together, these results suggest that the activation of TLR7 is crucial for the development of Th1 responses following *L. donovani* infection.

Since TLR7 not only signals through IRF5 but also through IRF7 [31] and IRF7 has recently been shown to regulate killing of intracellular *Leishmania* in marginal zone macrophages [32], we wanted to determine whether IRF7 contributed to the defect in Th1 responses we observed in *Tlr7^-/-^* mice. Hence, we infected *Irf7^-/-^* mice with *L. donovani* amastigotes and compared the development of Th1 responses in these mice to infected WT controls. The results revealed that in *Irf7^-/-^* mice, *L. donovani* induced IFNγ-producing CD4^+^ T-cells at frequencies comparable to infected WT mice (Fig. 7C and D).

Finally, we compared the cell-specific IRF-5 mRNA expression pattern in WT and *Tlr7^-/-^* mice at d14 and d28 p.i.. As previously shown (Fig. 6), only the CD5^+^ fraction (T-cells) had upregulated IRF-5 mRNA in the spleen of infected WT mice (Fig. 7E). The IRF-5 mRNA expression was not significantly higher in B-cells and non-B/non-T-cells from infected WT mice compared to the corresponding naïve WT cell populations (data not shown). Interestingly, we could not detect any upregulation of IRF-5 mRNA expression in splenic T-cells from infected *Tlr7^-/-^* mice at d28 p.i., suggesting that TLR7 is essential for the induction of IRF-5 in T-cells in the spleen at later stages of infection. In contrast, in the liver, IRF-5 mRNA expression was only upregulated in T-cells purified at day 14 p.i. from infected WT and *Tlr7^-/-^* mice (Fig. 7F). This suggests that increased IRF-5 expression in T-cells is independent of TLR7 during the early stages of infection in the liver. No IRF-5 mRNA could be detected at d28 p.i. in the liver of WT and *Tlr7^-/-^* mice (Fig. 7F).

Taken together, these data suggest that IRF-5 is a key molecular switch for the development of Th1 responses following *L. donovani* infection, and that TLR7-mediated IRF-5 activation plays a critical role during chronic infection.

## Discussion

In the present study we have demonstrated that the TLR7-mediated activation of IRF-5 is required for the development of host-protective Th1 responses to *L. donovani* at later stages of infection. Moreover, IRF-5 deficiency resulted in an IL-4 dominated response, reduced iNOS expression, and failure to control parasite growth in the liver. To our knowledge, the role of IRF-5 in modulating adaptive T-cell responses is a novel and previously undescribed finding.

One of the key points in the induction of a protective immunity to *Leishmania* parasites is the generation of IFNγ-producing CD4^+^ T-cells. Although IL-12 production by DC is crucial for the development of Th1 cells [25], the mechanism leading to the generation of these responses remains elusive. A study in *Myd88^-/-^* mice infected with *Leishmania* has highlighted the importance of TLRs, IL-1 and/or IL-18 in the induction of IL-12 and the generation of Th1 responses. Indeed, MyD88 deficiency resulted in complete abrogation of IFNγ production by CD4^+^ T-cells and an inability to control infection [5]. More recently, TLR9 activation was shown to play a crucial role in the induction of IL-12 secretion by DCs following *T. cruzi* and/or *Leishmania* infections [6], [7]. However, unlike *T. cruzi* infections, attenuation of Th1 responses to *Leishmania* infection in *Tlr9^-/-^* mice was only transient [7], [9]. These studies suggest that the regulation of Th1 responses in *Leishmania* infections is not exclusively mediated by TLR9 and might be governed by other pathogen recognition pathways as well. Our study pinpoints IRF-5, a transcription factor in the MyD88-mediated TLR pathways, as an essential factor in the development of adaptive Th1 responses following *Leishmania* infection. Interestingly, while Th1 responses in *Irf5^-/-^* mice were severely impaired 4 weeks after infection, expression of IRF-5 was not required during the first 2 weeks of infection, since the frequency of IFNγ-producing CD4^+^ T-cells in *Irf5^-/-^* mice was comparable to WT mice. This observation suggests that IRF-5 is not essential for the early induction of Th1 responses but is crucial for their further development and expansion. Although IRF-5 has been shown to have a major impact on the innate immune response to viral infections [17], [18], its role in shaping the development of adaptive Th1 responses has not been previously demonstrated.

Defective Th1 responses were also observed in *Tlr7^-/-^* mice infected with *L. donovani*. IRF-5 and IRF-7 are both known to be activated by the MyD88-dependant TLR7 signaling pathway [16], [31], however IRF-7 deficiency did not result in defective Th1 responses to *L. donovani*. TLR7 has been shown to recognize single-stranded RNA [33], [34] and to play an essential role in the immunity to ssRNA virus [24], [35]. Recognition of a DNA virus, the murine cytomegalovirus, by TLR7 has also been reported [36]. To date there is no indication of TLR7 activation by parasites.

The analysis of cell-specific IRF-5 mRNA expression pattern revealed that the only cells that upregulated IRF-5 during *L. donovani* infection at d14 and 28 p.i. were T-cells. This does not exclude, though, that other cell types such as DCs and macrophages may express IRF5 during earlier stages of infection. Nevertheless, IRF-5 expression in T-cells differed between the liver and the spleen: in the liver IRF5 expression is upregulated during the first 2 weeks of infection in a TLR7-independent manner; in contrast, in the spleen the upregulation occurs 4 weeks into the infection and appears to be mediated by TLR7. Since the defect in the Th1 responses seems to mainly affect the hepatic infection, at least until d28 p.i., and IRF-5 only appears to be upregulated in T-cells in the spleen during chronic infection, it is tempting to speculate that effector Th1 cells generated in the spleen are required for controlling parasite growth in the liver. Future experiments involving splenectomized mice should be able to prove this hypothesis.

It is possible that the upregulation of IRF-5 in T-cells may be required depending on the subsets and activation status of the cells. A role for IRF-5 in T-cells is yet unknown. Whether IRF-5 expression by T-cells is directly mediated by TLR 7/9 triggering or indirectly induced by Type I IFN, produced by APC following TLR7/9 signaling, is still an open question. Recent studies have highlighted a role for MyD88 in T-cells [37], [38], supporting the possibility of a direct TLR7/9 –mediated IRF-5 induction. Mice with MyD88 deficient T-cells only develop defective Th1 responses in a *Toxoplasma gondii* model [37], suggesting that MyD88 signaling in T-cells is essential for Th1 cell development. Human T-cells purified from HIV [39] and from Hepatitis C [40] patients were also shown to express TLR7 and/or TLR9. Why TLR7/IRF-5 activation is only required at later stages of infection and what role it plays in various T-cell subsets are two more questions that remain yet to be answered. Future investigations will address these questions using T-cell-specific IRF-5 and TLR7 knockout mice.

Interestingly, the severe defect in Th1 responses seen in *Irf5* ^-/-^ mice affected the hepatic but did not seem to have any effect on the splenic parasite burden, at least until day 28 p.i.. This is in agreement with a study by Engwerda and colleagues who have demonstrated that IL-12 neutralization did exacerbate infection in the spleen during the first 4 weeks of infection, even though IFNγ production, which is mainly derived from CD4^+^ T-cells, was severely reduced [2]. Thus, in contrast to the liver, Th1 responses are not critical for controlling parasite growth in the spleen during the first 28 days of infection. These observations imply that requirements for protection against *L. donovani* in the liver and in the spleen are very distinct, and underline again the organ-specific nature of the immune response during VL.

IRF-5 deficiency also resulted in a dramatic decrease in the extent of the inflammatory cell infiltration in the liver at day 28 p.i.. Moreover, *L. donovani* failed to induce splenomegaly in *Irf5^-/-^* mice, which is characteristic for *L. donovani* infection in WT mice. Since TLR7 deficiency did not significantly impair the recruitment of inflammatory cells to the liver of *L. donovani* infected mice, we can assume that there is some redundancy between different TLRs recognizing different PAMPs, but commonly utilizing IRF-5 in the induction of the inflammatory response during VL. Such overlapping effects between TLR7 and TLR9 have been observed during murine cytomegalovirus infection [36]. However, TLR7 and TLR9 were recently shown to have distinct effects in a murine model of Lupus [41] and also during experimental West Nile Encephalitis, where *Tlr7^-/-^* mice, but not *Tlr9^-/-^* mice, showed an impaired CD45^+^ leukocyte and macrophage infiltration at the site of infection [24]. Failure of these cells to migrate to infected target organs was caused by a significant reduction in IL-23 responses [24]. In *L. donovani* infected *Irf5^-/-^* mice, the level of IL-23 p19 were slightly higher compared to WT mice, suggesting that the lack of lymphocyte infiltration in the liver was not caused by the decreased induction of IL-23, but by some other yet unidentified pathways. Furthermore, the frequency of IL-17 producing cells, which are typically induced by IL-23 [42], was comparable in both groups of mice. This indicates that IRF-5 deficiency only affected the development of Th1 responses, but not the generation of Th17 cells in *L. donovani* infected mice.

NO is an important leishmanicidal effector molecule. It has direct microbial toxicity and it is also involved in the regulation of cytokine gene expression and cytokine responsiveness. NO is typically produced by classically activated macrophages upon triggering of interferon and TLR pathways that enhance expression of iNOS [28], [29], [30]. Expression of iNOS in mice infected with *L. donovani* appears to be tissue-specific: this enzyme is induced in the liver, however only limited iNOS expression is observed in the spleen [43]. Despite defective IFNγ responses in *Irf5^-/-^* mice the level of iNOS mRNA expression was comparable to the WT control group. Since iNOS expression can also be triggered by TLR pathways, high levels of iNOS mRNA in *Irf5^-/-^* mice may most likely be due to the high parasite burden in the liver at d28pi. Interestingly though, iNOS protein was not detected in the livers of *Irf5* ^-/-^ mice. A possible explanation for the lack of iNOS protein in *Irf5^-/-^* mice is that iNOS may be competing with arginase 1. This enzyme is commonly linked to alternative pathway of macrophage activation [44]. However, arginase 1 can also be induced in classically activated macrophages and can reduce NO production through competition with iNOS for their common substrate arginine [45]. Arginase 1 was also shown in vitro to suppress translation and enzymatic activity of iNOS without affecting mRNA levels [46], [47]. Nevertheless, arginase 1 mRNA was only slightly increased in *Irf5^-/-^* mice compared to WT mice, an increase that is probably not sufficient to inhibit translation of iNOS protein. Another possible explanation is that the severe defect in pro-inflammatory and the concomitant increase in Th2 responses observed in *Irf5^-/-^* mice may be responsible for the lack of amplification of the iNOS expression. Further investigations are needed to clarify the role of IRF-5 in the molecular mechanisms involved in the regulation of iNOS production.

In conclusion, we have indentified IRF-5 as a critical component for the development of Th1 responses to *Leishmania* infection. Furthermore, IRF-5 is an essential factor for the maintenance of the inflammatory response and plays a role in the indirect regulation of iNOS expression during *L. donovani* infection. Further experiments have yet to determine the molecular mechanism by which IRF-5 affects the development of host protective immunity to *L. donovani*.

## Materials and Methods

### Mice and parasites

Ethics statement: All experiments were approved by and conducted in accordance with guidelines of the Animal Care and Use Committee of the Johns Hopkins University School of Medicine.

C57BL/6J mice were obtained from The National Cancer Institute (Frederick, MD, USA), and B6.129S7-*Rag1^tm1Mom^*/J from The Jackson Laboratory. All mice were housed in the Johns Hopkins University animal facilities (Baltimore, MD) under specific pathogen-free conditions and used at 6–8 weeks of age. *Irf*5^-/-^ mice were a generous gift from Dr. T. Mak (University of Toronto, Canada) and were backcrossed to C57BL/6J for at least 10 generations. The SNP analysis confirmed that they are 100% C57BL/6. We have previously reported age related splenomegaly in *Irf*5^-/-^ mice that was associated with changes in splenic architecture [48]. The background of these mice was only 92% C57BL/6. In this study we have used 6–8 weeks old *Irf*5^-/-^ mice that have normal spleen size and splenic architecture. *Tlr7^-/-^* mice were a generous gift from Dr S. Akira (Osaka University, Japan). *Tlr7^-/-^*were backcrossed to C57BL/6J for 9 generations. *Irf7^-/-^* mice were a kind gift from Dr. T. Taniguchi (University of Tokyo, Japan). *Irf7^-/-^* were backcrossed to C57BL/6J for 8 generations. *Leishmania donovani* (strain LV9) parasites were maintained by serial passage in B6.129S7-*Rag1^tm1Mom^*/J mice, and amastigotes were isolated from the spleens of infected animals. Mice were infected by injecting 2×10^7^ amastigotes intravenously via the lateral tail vein. Hepatic and splenic parasite burdens were determined by examining methanol-fixed, Giemsa stained tissue impression smears. Data are presented as Leishman Donovan Units (LDU).

### Flow cytometry

Mice were euthanized at indicated time points. Mononuclear cells were purified from the liver as previously described [49]. Hepatic mononuclear cells and splenocytes were restimulated for 2 h at 37°C in the presence of bone marrow derived dendritic cells (BMDC) previously pulsed with paraformaldehyde fixed amastigotes. Brefeldin A was then added for a further 4 h. Cells were then stained with biotinylated anti-CD3 followed by PerCP-conjugated strepatvidin, FITC-conjugated anti-CD4, and APC-conjugated anti-IFNγ (all BD Bioscience). Flow cytometric analysis was performed with a LSRII flow cytometer (Becton Dickinson). 350,000 cells per sample were acquired and analyzed with the FACSDiva software.

### Cell sorting

Spleens from infected and naive C57BL/6 mice were divided into 2 groups. Group A: B-cells were first enriched from splenic single cell suspension using anti-B220 beads (Miltenyi Biotec) following manufacturer instructions. B-cells were then sorted to >98% purity using FACSVantage (Becton Dickinson) based on their expression of CD19 and B220. The B220 negative fraction was then incubated with anti-CD5 beads for the enrichment of T-cells. T-cells were then sorted to >98% purity using FACSVantage based on their expression of CD4^+^, CD8^+^ and NK1.1^-^. Group B: splenocytes were first incubated with anti-CD11c beads and conventional splenic DCs were then sorted to >98% purity based on their expression of CD11c and MHCII. The CD11c negative fraction was then incubated with anti-CD11b beads. CD11b+ cells were sorted into different populations based on their expression of Gr1, MHCII and CD11b.

Livers from infected and naïve C57BL/6 mice and from infected *Tlr7^-/-^* mice were incubated with anti-CD5 beads in order to enrich T-cells. The purity of the T-cell preparation was >87%. Spleens from infected WT and *Tlr7^-/-^* mice were first incubated with anti-B220 beads; the B220 negative fraction was then incubated with anti-CD5 beads. >82% of the B220+ cells were B-cells; >94% of the CD5+ cells were T-cells.

### Preparation and infection of bone marrow derived macrophages

Macrophages were differentiated from the bone marrow following red blood cell lysis. Cells were incubated with 30 ng/ml M-CSF (RnDSystems) for 5 days. Prior to use cells were counted and seeded at the 5×10^5^ cells/well in a 24 well plate, rested in the absence of M-CSF for several hours and then treated with LPS (1 µg/ml) (Sigma-Aldrich) or *L. donovani* (MOI 10) for 1 h. Media was then changed to remove any extracellular parasites and cells incubated in fresh media for a further 18 h. At experimental end point supernatant was collected and cells washed in PBS and lysed for RNA extraction.

### RNA extraction and real time PCR analysis

For the analysis of the IL-12p35/IL-12p40 mRNA induction, WT and *Irf5* ^-/-^ mice were infected with 2×10^7^ *L.donovani* amastigotes. 5 h later, mice were euthanized and CD11c^+^ dendritic cells were isolated from splenic single cell suspensions using anti-CD11c beads (Miltenyi Biotec) following manufacturer instructions. The purity of the preparation was about 85%. RNA was extracted using Trizol (Invitrogen) as per manufacturer' instructions. For purified cell populations listed in the previous section RNA was extracted using the RNEasy Mini Kit (Qiagen). Reverse transcription was performed using the QuantiTect Reverse Transcription kit (Qiagen). SybrGreen was used to assay beta actin [17], IL-12p40 and IL-12p35. The following primers were used: IL-12p35 F- CCACCCTTGCCCTCCTAAAC and R-GGCAGCTCCCTCTTGTTGTG; IL-12p40 F- CTTGCAGATGAAGCCTTTGAAGA and R- GGAACGCACCTTTCTGGTTACA. For infected mice, livers and spleens were collected at indicated time points and whole tissue RNA extracted using Trizol, cDNA generated using the QuantiTect Reverse Transcription kit. Other than IL-12p35 and IL-12/23p40 all gene expression was analyzed using Taqman primers with a StepOnePlus cycler (Applied Biosystems).

### NO quantiation

Nitric oxide was quantitated using the Greiss assay (Promega) according to manufacturer's instructions.

### Immunohistochemistry

For frozen sections, livers from infected mice and uninfected controls were embedded in OCT (TissueTek), snap frozen and 5 µm sections cut. Sections were stained for iNOS/NOS2 using an anti-iNOS polyclonal antibody generated in rabbit (Chemicon/Millipore) and detected using anti-rabbit IgG conjugated to HRP and DAB substrate (both Vector Laboratories). Paraffin-embedded sections were prepared from liver fixed in neutral buffered formalin, cut to 5 µm and stained by H&E. All immunohistochemistry was analyzed by light microscopy and photographs taken at the indicated magnifications.

### Luciferase assay

HEK293 cells were seeded at 1×10^5^ cells/well in 96 well plates. Cells were transfected with the *NOS2*-luc reporter construct (50 ng) (Addgene) and Renilla luciferase (10 ng) with either MyD88 (50 ng) and/or murine IRF-5 (100 ng) expression plasmids. 24 hours after transfection cells were lysed and assayed for luciferase activity using the Promega Dual-luciferase reporter assay. Luciferase activity was normalized against Renilla. Data shown is the mean± SEM for triplicate samples from 2 independent experiments.

### Statistical analysis

Results were analyzed using an unpaired Student *t*-test. *P*<0.05 was considered significant. Real time PCR results were analyzed using an unpaired Student *t*-test or the Mann-Whitney test. For bone marrow derived macrophages the non parametric *t*-test with Welch's correction was used. *P*<0.05 was considered significant. Experiments were repeated at least twice.

## Supporting Information

Figure S1 (A) The percentage of Gr1^hi^ CD11b^hi^ MHCII^-^ cells (neutrophils) in the spleen of WT and *Irf5* ^-/-^ mice at day 14 and 28 p.i. was determined by flow cytometry. Data is shown as the mean ± SEM. Flow cytometry data is representative of two independent experiments. * denotes *p*<0.05. (B) Representative sections of H&E stained, paraffin-embedded spleens from WT and *Irf5* ^-/-^ mice. Pictures were taken at indicated magnifications and show the presence of a large neutrophil infiltrate in the spleen of d28 infected *Irf5* ^-/-^ mice as indicated by arrows.
(7.54 MB TIF)

Figure S2 Bone marrow derived macrophages were incubated in vitro with *L. donovani* (MOI 10) or LPS (1 µg/ml) for 18 h. (A) Nitric oxide production in the culture supernatant measured by Griess assay. (B) Real time PCR analysis measuring mRNA for IL-6, TNFα, and IFNβ. All data is presented as the mean ± SEM.
(0.18 MB TIF)

Figure S3 Representative scatter plots from WT and *Tlr7* ^-/-^ mice infected with *L. donovani* showing IFNγ production by CD4^+^ T cells in the liver at different times post infection. Plots are representative of 2 independent experiments.
(0.22 MB TIF)
