# Supplementary figures and images for: Critical Role of IRF-5 in the Development of T helper 1 responses to Leishmania donovani infection
Source: PLoS Pathog. 2011 Jan 6;7(1):e1001246. doi: 10.1371/journal.ppat.1001246 (PMC3017120; doi:10.1371/journal.ppat.1001246)

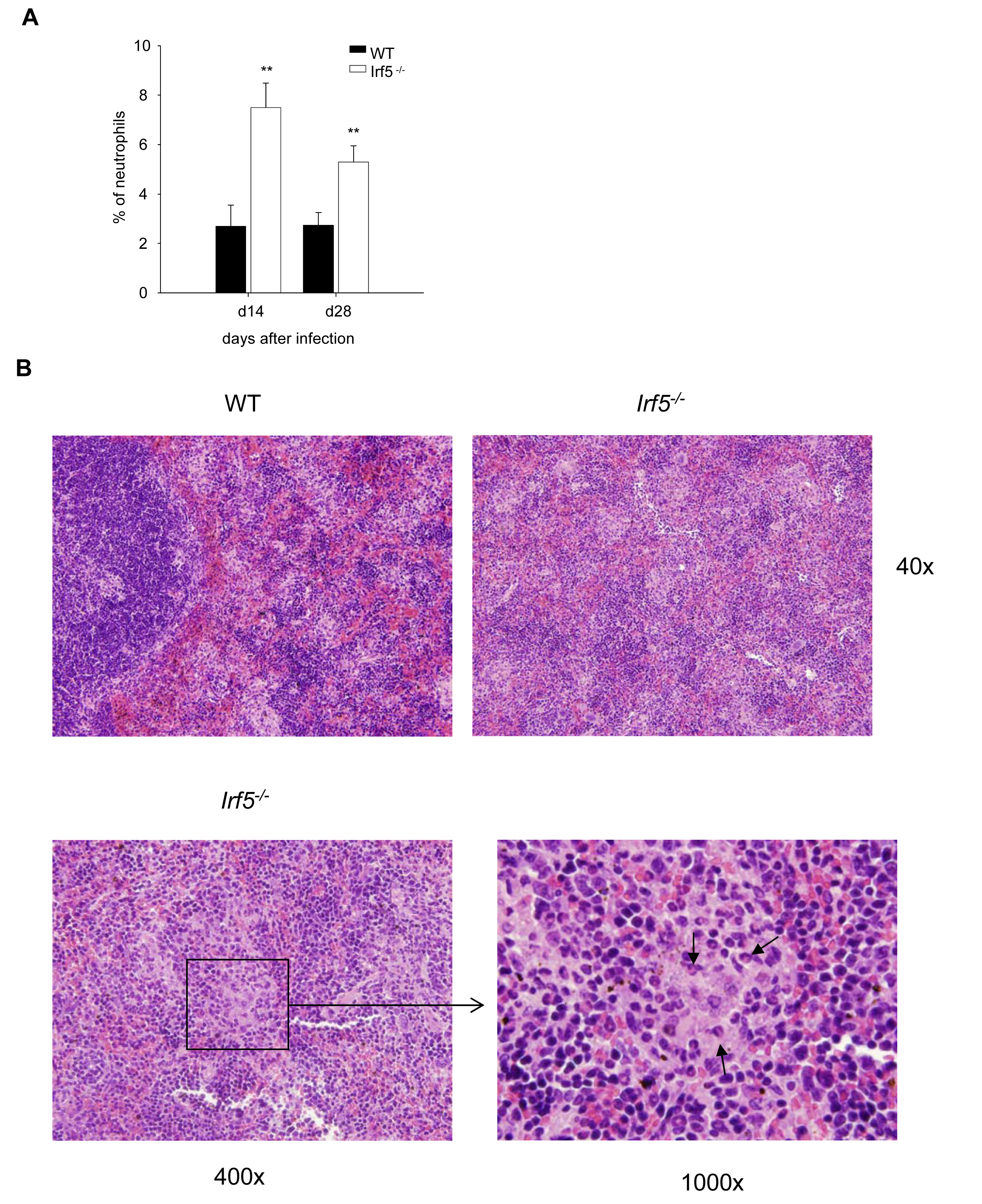

Supplement: Figure S1 — (A) The percentage of Gr1hi CD11bhi MHCII- cells (neutrophils) in the spleen of WT and Irf5 -/- mice at day 14 and 28 p.i. was determined by flow cytometry. Data is shown as the mean ± SEM. Flow cytometry data is representative of two independent experiments. * denotes p<0.05. (B) Representative sections of H&E stained, paraffin-embedded spleens from WT and Irf5 -/- mice. Pictures were taken at indicated magnifications and show the presence of a large neutrophil infiltrate in the spleen of d28 infected Irf5 -/- mice as indicated by arrows. (7.54 MB TIF) [file ppat.1001246.s001.tif]

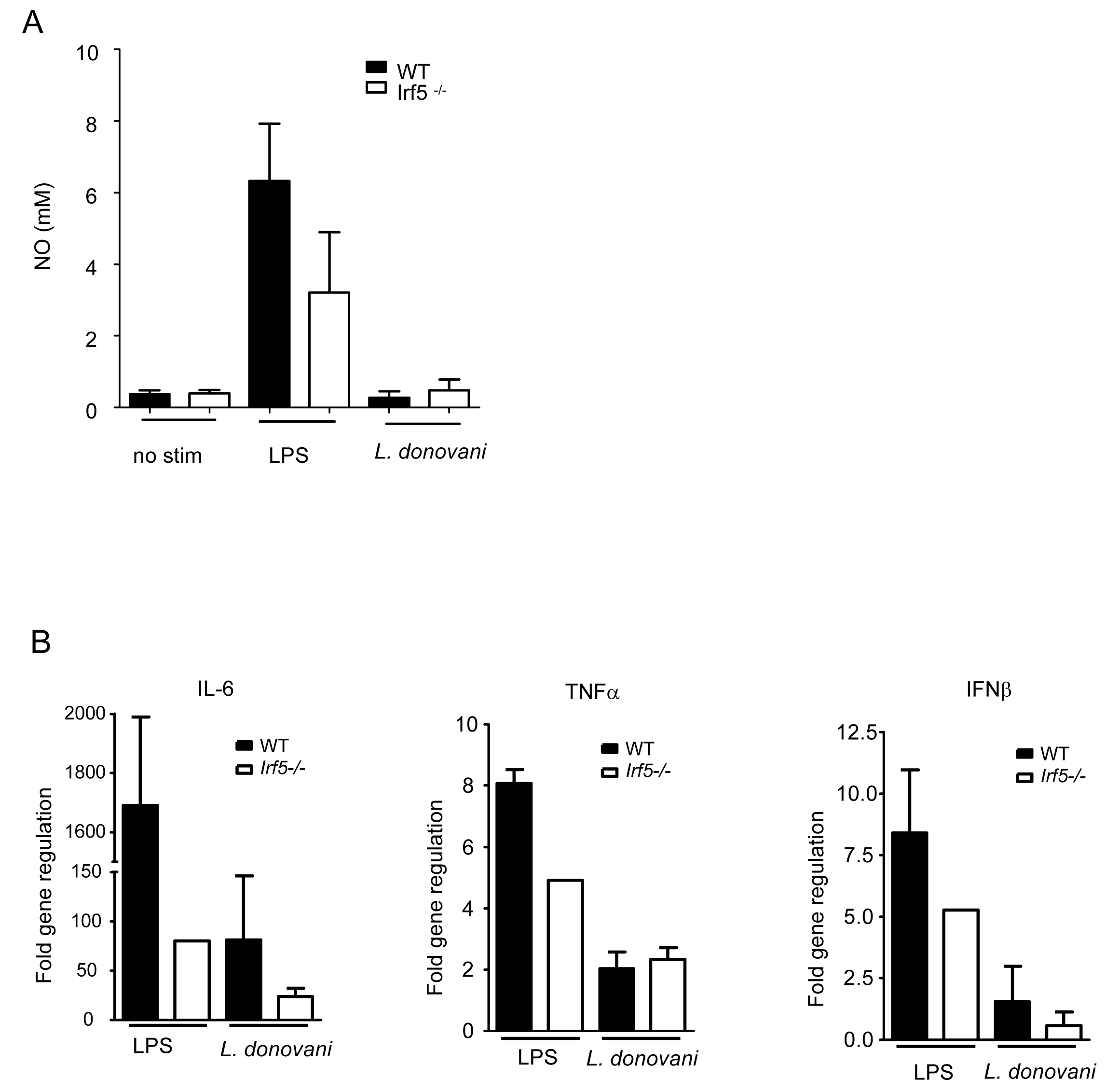

Supplement: Figure S2 — Bone marrow derived macrophages were incubated in vitro with L. donovani (MOI 10) or LPS (1 µg/ml) for 18 h. (A) Nitric oxide production in the culture supernatant measured by Griess assay. (B) Real time PCR analysis measuring mRNA for IL-6, TNFα, and IFNβ. All data is presented as the mean ± SEM. (0.18 MB TIF) [file ppat.1001246.s002.tif]

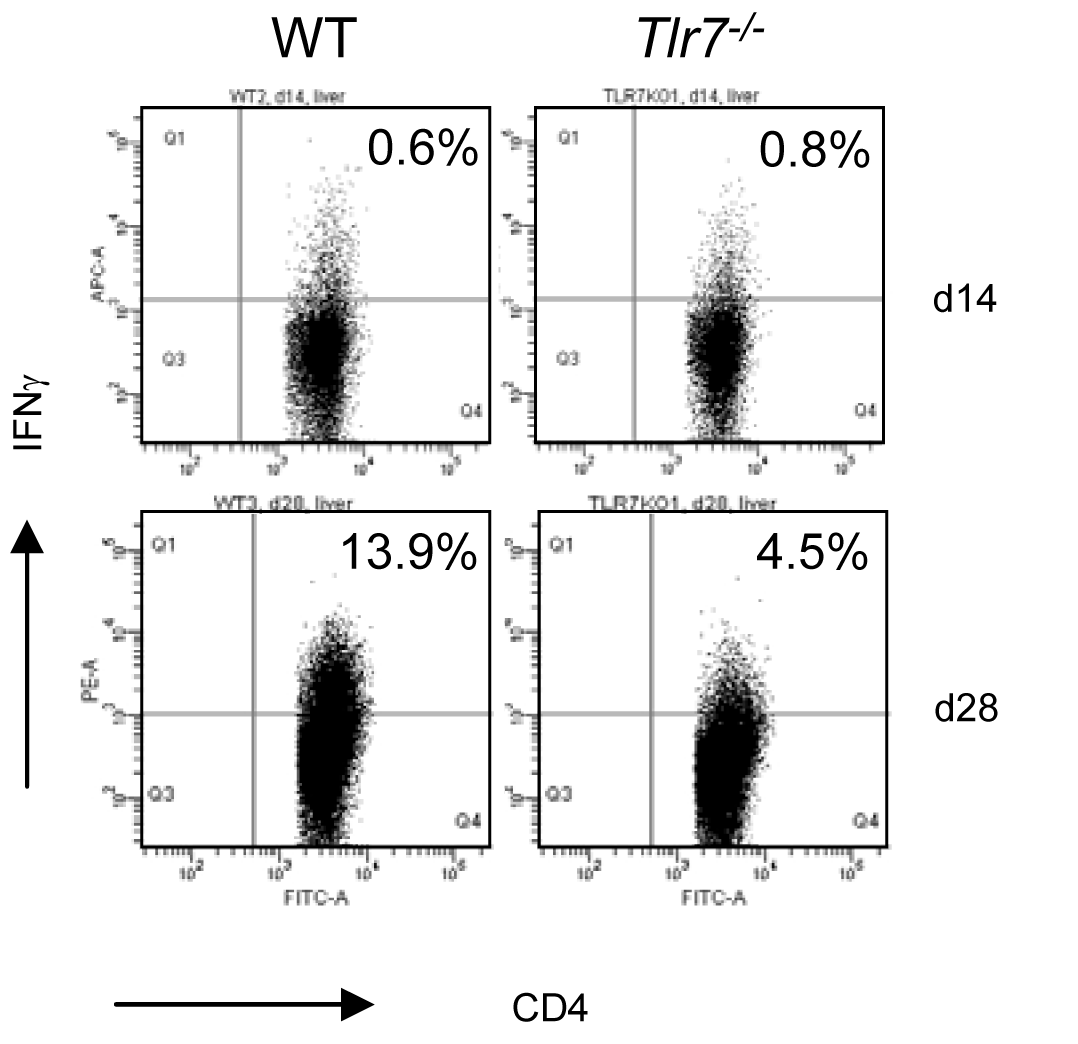

Supplement: Figure S3 — Representative scatter plots from WT and Tlr7 -/- mice infected with L. donovani showing IFNγ production by CD4+ T cells in the liver at different times post infection. Plots are representative of 2 independent experiments. (0.22 MB TIF) [file ppat.1001246.s003.tif]
